# Robinson’s Fibrous Filling

**Published:** 1884-09

**Authors:** 


### ROBINSON'S FIBROUS FILLING.

Among all the varied new preparations for filling teeth, that have within a comparatively recent period been offered to the profession, there is none that seems to have met with more general favor than Robinson's Tin. "Uncle Jerry" was so widely known and so generally esteemed, that when he announced a good thing it immediately challenged the attention of a large part of the profession. We think his Fibrous Filling has come to stay. It presents so many advantages that no intelligent operator can afford to be without it. It is so perfectly adaptable to the cervical walls of large cavities, and gold unites with it so readily, that the dentist who does not make use of it in such localities is missing a good thing. Try it; you who desire to reach the highest point in operative dentistry.

